# Morphological and Molecular Characterization, and Demonstration of a Definitive Host, for *Sarcocystis masoni* from an Alpaca (*Vicugna pacos*) in China

**DOI:** 10.3390/biology11071016

**Published:** 2022-07-06

**Authors:** Zhipeng Wu, Jun Sun, Junjie Hu, Jingling Song, Shuangsheng Deng, Niuping Zhu, Yurong Yang, Jianping Tao

**Affiliations:** 1 Yunnan Key Laboratory for Plateau Mountain Ecology, Restoration of Degraded Environments, School of Ecology and Environmental Sciences, Yunnan University, Kunming 650091, China; wzp@mail.ynu.edu.cn (Z.W.); sjmrxsm@mail.ynu.edu.cn (J.S.); 2 Electron Microscope Laboratory, Kunming Medical University, Kunming 650500, China; ynkmsongjingling@sina.com; 3 School of Biological Sciences, Yunnan University, Kunming 650091, China; ssdeng@ynu.edu.cn; 4 College of Veterinary Medicine, Henan Agricultural University, Zhengzhou 450064, China; zhuniuping414@163.com; 5 College of Veterinary Medicine, Yangzhou University, Yangzhou 225009, China; yzjptao@126.com

**Keywords:** *Sarcocystis masoni*, alpaca, morphological and molecular characterization, life cycle

## Abstract

**Simple Summary:**

*Sarcocystis* spp. are cyst-forming intracellular protozoan parasites characterized by a two-host prey–predator life cycle. The alpaca (*Vicugna pacos*) is one of the South American camelids (SACs), and in recent years, this animal was introduced to China to be raised for its meat, skin, and wool and to be kept as tourist attractions and as pets. There is considerable confusion regarding the classification and nomenclature of the species of *Sarcocystis* in SACs. Two *Sarcocystis* species, named *S. auchenia* and *S. masoni*, are currently regarded as valid in SACs based on sarcocyst morphology and *18S* rDNA sequences. However, the definitive host of *S. masoni* remains unknown. Here, *S. masoni* sarcocysts in an alpaca were morphologically described and molecularly characterized. Furthermore, the life cycle of *S. masoni* was completed via experimental animal infection. The present analysis showed that *S. masoni* has a close relationship with *S*. *cameli* in the dromedary camel (*Camelus dromedaries*), and the relationship between the two parasites needs to be clarified in the future.

**Abstract:**

Only *18S* rDNA sequences of *Sarcocystis* spp. in South American camelids (SACs) are deposited in GenBank as references, and the definitive host of *S. masoni* in SACs is still unclear. Here, *S. masoni* sarcocysts detected in an alpaca (*Vicugna pacos*) in China were investigated with the aid of light (LM) and transmission electron (TEM) microscopy, and characterized using four genetic markers, i.e., *18S* rDNA, *28S* rDNA and *ITS*, and the mitochondrial *cox*1. Additionally, the life cycle of the parasite was completed via experimental animal infection. Under LM, *S. masoni* sarcocysts exhibited numerous 1.3–2.1 μm conical protrusions. Under TEM, the sarcocyst wall contained conical, cylindrical, or irregular-shaped villar protrusions, similar to type 9j. Two dogs (*Canis familiaris*) fed *S. masoni* sarcocysts shed sporocysts with a prepatent period of 8–9 days. The newly obtained *18S* rDNA sequences showed 98.4–100% identity with those of *S. masoni* in SACs previously deposited in GenBank. Interestingly, the newly obtained sequences of *18S* rDNA and mitochondrial *cox*1 shared 99.6–100% and 98.2–98.5% identity, respectively, with those of *S*. *cameli* in dromedary camels (*Camelus dromedaries*). Phylogenetic analysis based on sequences of *18S* rDNA, *28S* rDNA, or mitochondrial *cox*1 revealed that *S. masoni* has a close relationship with *Sarcocystis* spp. in ruminants. The relationship between *S. masoni* and *S*. *cameli* deserves to be further clarified in the future.

## 1. Introduction

*Sarcocystis* spp. are cyst-forming intracellular protozoan parasites with an obligate two-host life cycle, with predators as definitive hosts and their prey animals as intermediate hosts. Collectively, these species have considerable veterinary, economic, and public health importance [1]. The alpaca (*Vicugna pacos*, formerly *Lama pacos*) is one of the South American camelids (SACs). This animal is a domesticated farm animal and is mainly distributed in Peru, where it plays a crucial role in local socioeconomics [2]. During the last two decades, this animal has been introduced to China to be raised for its meat, skin, and wool and to be kept as tourist attractions and as pets [3].

SACs, including the alpaca, llama (*Lama glama*), vicuna (*Vicugna vicugna*), and guanaco (*Lama guanicoe*), serve as important intermediate hosts for *Sarcocystis* spp., which usually cause subclinical infections in SACs, although fatal cases have also been reported [4,5]. Two kinds of sarcocysts, macroscopic cysts (up to 8 mm long) and microscopic cysts (up to 800 µm long), have been observed and described in SACs. However, there is considerable confusion regarding the classification and nomenclature of the species of *Sarcocystis* in SACs [1]. The history of the taxonomy of *Sarcocystis* spp. in SACs has been reviewed by different authors recently [1,6,7]. Currently, based on the sarcocysts’ ultrastructure, the macroscopic cysts in SACs are regarded as *S*. *auchenia*, which was originally found in a llama and named by Brumpt (1913) [8], and the microscopic cysts in SACs are regarded as *S. masoni*, named by Moré et al. (2016) [6]. The microscopic cysts have been called *S*. *lamacenis*/*lamacanis* in two review papers [9,10], but no explanation for these proposals was provided by the authors.

Traditionally, sarcocyst structure and life cycle are the two major criteria for naming a new species of *Sarcocystis* in a given intermediate host [1]. The definitive host of *S*. *aucheniae* in guanaco and llama has been experimentally demonstrated to be dogs (*Canis familaris*) [11,12]. However, the life cycle of *S*. *masoni* remains unknown. At present, PCR assays and sequencing procedures are considered much more practical, accurate, and reliable for the delineation and identification of *Sarcocystis* species than traditional methods based on the morphological characteristics [13,14]. Only the *18S* rDNA sequences of *S*. *masoni* and *S*. *auchenia* presently deposited in GenBank serve as references for species identification. However, the discriminatory power of the gene has been shown to be unsuitable for the differentiation of closely related lineages of *Sarcocystis* in ruminants due to its highly conserved nature [13,15]

Therefore, the aims of the present study were: (1) to investigate the morphology of sarcocysts in an alpaca from China using light (LM) and transmission electron (TEM) microscopy; (2) to sequence and analyze the near-full or full length of the three nuclear DNA regions *18S* rDNA, *28S* rDNA, and *ITS* (*ITS*1-*5.8*S-*ITS*2) and the partial mitochondrial *cox*1 of the sarcocysts to augment the species description; and (3) to elucidate the *S. masoni* life cycle in the definitive host via experimental infection.

## 2. Materials and Methods

### 2.1. Examination for Sarcocysts in Alpaca

In December 2021, one captive-bred alpaca died from an illness at a zoo in Zhengzhou, the capital city of Henan province, China. The veterinarian conducted a pathological dissection, and the myocardium of the animal was shipped on ice to the zoological laboratory at the School of Ecology and Environmental Sciences, Yunnan University, for diagnosis of sarcocystosis. In the laboratory, 1–3 mm pieces were pressed between 2 glass slides and examined for sarcocysts via stereomicroscopy. Sarcocysts were extracted from muscular fibers and processed for LM and TEM, experimental infection, and DNA analysis. For TEM, six sarcocysts were fixed in 2.5% glutaraldehyde in cacodylate buffer (0.1 M, pH 7.4) and examined using a JEM100-CX TEM (JEOL Ltd., Tokyo, Japan) at 80 kV. For DNA isolation, individual sarcocysts were stored in sterile water at −20 °C prior to processing.

### 2.2. Experimental Infection of Potential Definitive Hosts

Three dogs and three cats (*Felis catus*) were used for the experimental infection. These animals were 1 to 2 months old and were purchased from a commercial source, housed separately in steel cages, and fed dog or cat pellets and water ad libitum. The feces of these animals were examined for four successive days with the use of centrifugal flotation and a sucrose solution (density 1.20 at 20 °C) before infection to prove that they were coccidian free.

Pieces of muscle from the alpaca were fed to two dogs and two cats. The remaining animals were kept as controls. Before inoculation, it was confirmed by extensive microscopic examination that the muscles of the alpaca contained only microcysts. The animals were each fed muscle pieces containing approximately 600 sarcocysts. Fecal samples from the cats and dogs were tested daily for at least 20 days post infection (PI) to determine the presence of sporocysts or oocysts. All animals were euthanized at 20 days PI. The small intestine of each animal was removed, and a mucosa sample from the small intestine, approximately 2.5 cm in length in each part, was examined for the presence of oocysts or sporocysts and via DNA extraction.

### 2.3. Molecular Characterization

Genomic DNA was extracted from six individual sarcocysts isolated from the alpaca and six mucosa samples each from the small intestine of the experimental animals using a TIANamp Genomic DNA Kit (Tiangen Biotech Ltd., Beijing, China) according to the manufacturer’s instructions. Four genetic markers—*18S* rDNA, *28S* rDNA, *ITS*, and mitochondrial *cox*1—were amplified from the sarcocysts using the primer pairs shown in Table 1.

To further verify the results of experimental infection, mitochondrial *cox*1 of the parasite in the definitive hosts was amplified and sequenced using the same primers SF1/SR9 for sequence analysis and polymerase chain reaction–restriction fragment length polymorphism (PCR-RFLP). PCR products were gel-purified, cloned and sequenced, and assembled using the methods detailed in a previous paper [20]. For PCR-RFLP, PCR products of mitochondrial *cox*1 obtained from sarcocysts and oocysts/sporocysts were digested separately with restriction enzymes ClaI and AvaII (New England BioLabs).

Phylogenetic analyses were conducted separately on the nucleotide sequences of the *18S* rDNA, *28S* rDNA, and mitochondrial *cox*1 sequences using MEGAX software [21]. The maximum likelihood (ML) trees of *18S* rDNA, *28S* rDNA, and mitochondrial *cox*1 were generated using the Tamura 3-parameter, Hasegawa–Kishino–Yano, and Kimura 2-parameter models, respectively, according to the Find Best DNA/Protein Models program integrated into MEGAX. All sites were used. The reliability of the maximum likelihood phylograms was tested via the bootstrap method using 1000 replications.

The *18S* rDNA, *28S* rDNA, and mitochondrial *cox*1 sequences of *Sarcocystis* spp. from different hosts were downloaded from GenBank and aligned using the ClustalW program implemented in MEGAX, using a gap opening penalty of 10/10 and a gap extension penalty of 0.1/0.2 as pairwise and multiple alignment parameters, respectively. The alignments were subsequently checked visually; some sequences were slightly truncated at both ends, so that all sequences started and ended at the same nucleotide positions. The final alignment of the *18S* rDNA sequences consisted of 33 nucleotide sequences and 1592 aligned positions including gaps from 24 taxa. *Cystoisospora ohioensis* (GU292304), *Besnoitia besnoiti* (DQ227418), and *Toxoplasma gondii* (U03070) were chosen as outgroups. The final alignment of the *28S* rDNA sequences consisted of 26 nucleotide sequences and 3839 aligned positions including gaps from 24 taxa. *Hammondia heydorni* (AF159240), *B. besnoiti* (AF076900), and *T. gondii* (AF076901) were chosen as outgroups. The final alignment of mitochondrial *cox*1 sequences consisted of a total of 37 nucleotide sequences and 995 aligned positions with no gaps from 29 taxa. *T. gondii* (JX473253), *H. triffittae* (JX473247), and *H. heydorni* (JX473251) were used as outgroup species to root the tree.

## 3. Results

### 3.1. Morphological Observation of Sarcocysts in the Alpaca

**Figure 1:**
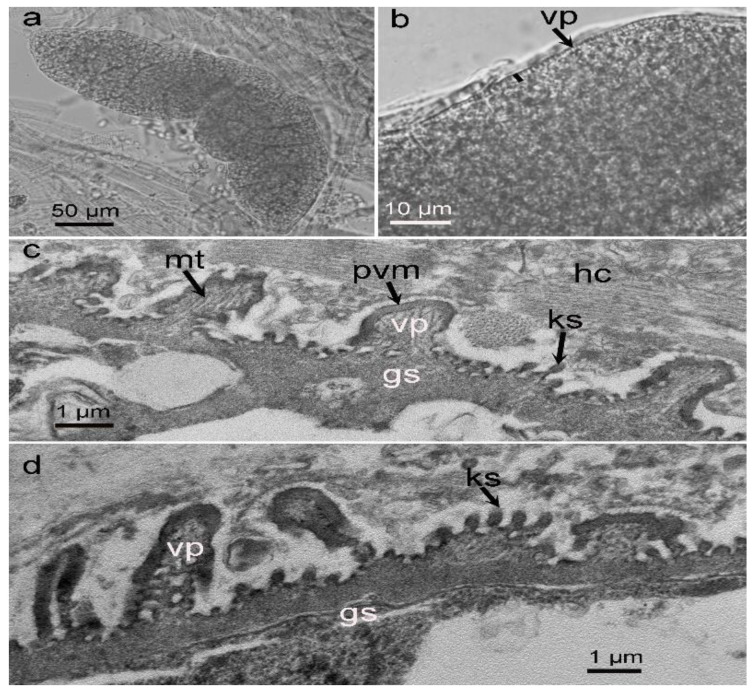
Morphological characteristics of *Sarcocystis masoni* sarcocysts isolated from the myocardium of an alpaca. (**a**) Overview of a sarcocyst (unstained, light microscopy, LM). (**b**) Sarcocyst bounded by short conical villar protrusions (vp) (unstained, LM). (**c**,**d**) Diagonal section of a sarcocyst (under transmission electron microscopy, TEM). Sarcocyst surrounded by host cell (hc). The sarcocyst wall exhibits numerous villar protrusions (vp) lined by an electron-dense parasitophorous vacuolar membrane (pvm). Knob-like structures (ks) present on the surface of the pvm. Each vp contains scattered microtubules (mt) in its core, which extend from the tip of the vp into the ground substance (gs).

Only sarcocysts resembling those of *S*. *masoni* were found in the alpaca. LM examination revealed that the sarcocysts were microscopic, measuring 342–550 × 88–102 μm (*n* = 20) in size, and exhibited numerous 1.3–2.1 μm conical villar protrusions (vp) (Figure 1a,b). They were septate and contained bradyzoites that were 10.2–12.3 × 2.8–5.5 μm (*n* = 20) in size.

Four sarcocysts from the alpaca were examined via TEM, all of which appeared to have walls that were ultrastructurally similar and that closely resembled “type 9j” (Figure 1c,d). The sarcocyst walls contained conical, cylindrical, or irregular-shaped villar protrusions (vp) depending on the plane of the section. The vp were at irregular distances and lined by a 60–80 nm thick electron-dense parasitophorous vacuolar membrane (pvm), with knob-like structures (ks) on the pvm. Each vp had scattered microtubules, which extended from the tip of the villus into the middle of the ground substance (gs). The gs, measuring 0.6–1.1 μm in thickness, was located immediately beneath the sarcocyst wall.

### 3.2. Infection of the Definitive Hosts

**Figure 2:**
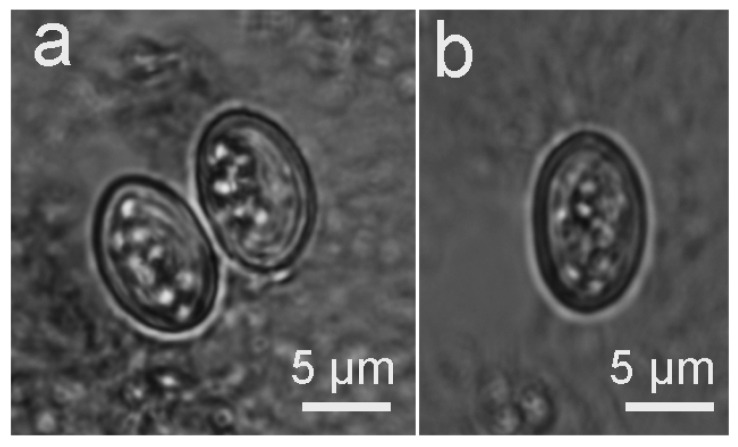
Light micrograph of *Sarcocystis masoni* sporulated oocyst and sporocysts in the feces of experimentally infected dogs (unstained). (**a**) Sporulated oocyst. (**b**) Sporocyst.

The two dogs that were fed with muscle tissues containing *S. masoni* sarcocysts from the alpaca both excreted sporulated oocysts and sporocysts (Figure 2a,b) beginning at Days 8 and 9 PI. The oocysts measured 12.5–15.8 × 9.8–11.2 μm (*n* = 10) with two elliptical sporocysts that measured 10.2–11.6 × 7.3–8.2 μm. Upon the death of the dogs and examination of their intestinal mucosa, the oocysts and sporocysts were found to be mostly concentrated toward the tips of the villi, but some were located deep in the villi as well. However, the intestinal mucosa was not appreciably altered, and infected dogs did not show any clinical signs due to sarcosporidian infection. No oocysts or sporocysts were found in the three cats or in the control dog.

### 3.3. Molecular Analysis

The four selected genes (*18S* rDNA, *28S* rDNA, *ITS*, and mitochondrial *cox*1) and mitochondrial *cox*1 alone were successfully amplified, sequenced, and assembled from six individual sarcocysts in the alpaca and from oocysts in the intestines of the two infected dogs, respectively. The six *18S* rDNA sequences (ON533528–ON533533) were 1848 and 1849 bp in length and shared 99.2–99.8% identity (average 99.5%). The most similar sequences in GenBank to the newly obtained *18S* rDNA sequences were those of *S*. *cameli* (OM462704 and OM462705) obtained from one-humped camels (*Camelus dromedaries*) (99.6–100% identity, average 99.8%) and *S*. *masoni* obtained from SACs, including 99.4–100% identity (average 99.6%) in alpacas (MW481703, MW481704, KU527112 and KU527113), 98.4–99.6% identity (average 99.0%) in guanacos (KU527107–KU527109), and 99.5–99.7% identity (average 99.6%) in llamas (KU527110 and KU527111), followed by those of *S*. *bovini* (93.5–94.0% identity, average 93.7%) in cattle (*Bos taurus*) (KT901139–KT9011). Nevertheless, the identity with those of *S*. *aucheniae* was only 87.1–91.8% (average 90.5%) in SACs (KU527114–KU527124).

The six *28S* rDNA sequences (ON533536–ON533541) were 3404–3414 bp in length and shared 98.7–99.6% identity (average 99.1%). The most similar sequences in GenBank to the newly obtained *28S* rDNA sequences were those of *Sarcocystis* spp. obtained from domestic ruminants, including *S*. *gigantea* (U85706) in sheep (*Ovis aries*) (91.9–92.2% identity, average 92.1%), *S*. *capracanis* (AF012885, KU820978, KU820979) in goat (*Capra hircus*) (91.5–91.6% identity, average 91.6%), and *S*. *cruzi* (AF076903) in cattle (91.5–91.6% identity, average 91.6%).

The six *ITS* sequences (ON540302–ON540307) were 1547–1566 bp in length and shared 92.7–98.4% identity (average 94.8%). BLAST searches indicated that no sequences in GenBank shared significant similarities with the newly obtained *ITS* sequences.

The six mitochondrial *cox*1 sequences obtained from the sarcocysts were 1085 bp in length and shared 99.4–100% identity (average 99.6%). Therefore, only five sequences (ON564410–ON564414) were deposited in GenBank. The four mitochondrial *cox*1 sequences obtained from oocysts in the two dogs were 1085 bp in length and shared 99.7–100% identity (average 99.8%). Therefore, only three sequences (ON564415–ON564417) were deposited in GenBank. The similarity between sarcocysts and oocysts was 99.0–100% (average 99.5%). The most similar sequence in GenBank was that of *S*. *cameli* (MW651858) in the one-humped camel (98.2–98.5% identity, average 98.4%), followed by those of *S*. *rommeli* (KY10286–KY10292) in cattle (79.5–80.5% identity, average 80.3%).

### 3.4. PCR-RFLP Based on Mitochondrial cox1 Obtained from S. masoni Sarcocysts and Oocysts

**Figure 3:**
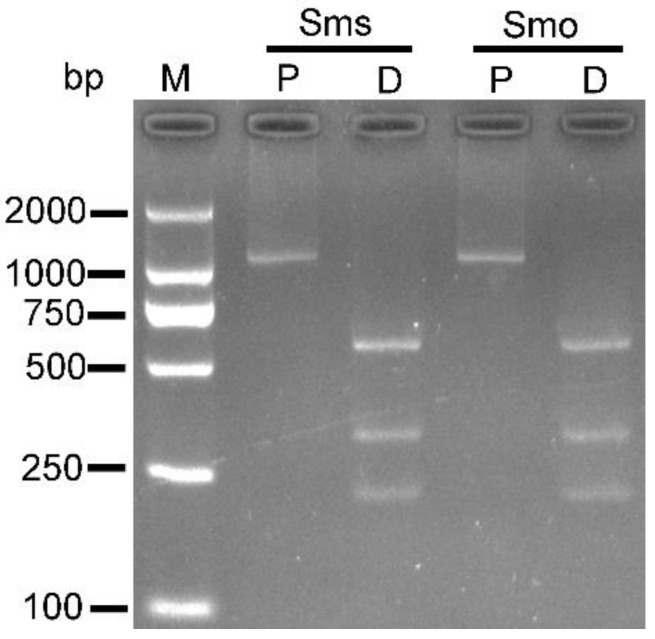
Results of PCR with primers SF1/SR9 and restriction enzyme digestion with ClaI and AvaII of sarcocyst and oocyst DNA from *Sarcocystis masoni*. M, molecular mass marker; Sms, *S*. *masoni* sarcocyst; Smo, *S*. *masoni* oocyst; P, PCR product; D, digestion of PCR product with ClaI and AvaII.

The PCR-amplified products (1085 bp) of mitochondrial *cox*1 from *S*. *masoni* sarcocysts and oocysts were successfully digested by ClaI and AvaII and produced three fragments (218, 316, and 551 bp) (Figure 3).

### 3.5. Phylogenetic Analysis

**Figure 4:**
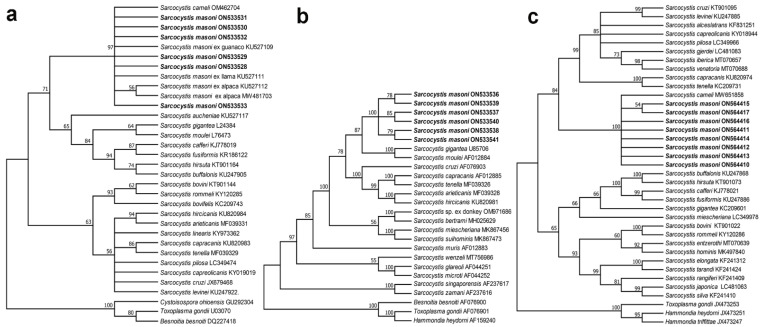
Phylogenetic trees of selected *Sarcocystis* species. The trees constructed based on (**a**) *18S* rDNA sequences, (**b**) *28S* rDNA sequences, and (**c**) mitochondrial *cox*1 sequences using maximum likelihood (ML) with the Tamura 3-parameter, Hasegawa–Kishino–Yano, and Kimura 2-parameter models, respectively. The values between the branches represent bootstrap values per 1000 replicates, and values below 50% are not shown. (**a**) The six newly obtained *18S* rDNA sequences of *S*. *masoni* (ON533528–ON533533, shown in boldface) obtained from the alpaca formed an individual clade with those of *S*. *cameli* in the one-humped camel and *S*. *masoni* in the llama, alpaca, and guanaco. (**b**) The six new sequences of *S*. *masoni* (ON533536–ON533541, shown in boldface) obtained from an alpaca formed an individual clade within a group comprising *Sarcocystis* spp. in domestic ruminants. (**c**) The eight sequences of *S*. *masoni* (ON564410–ON564417, shown in boldface) obtained from the alpaca and two infected dogs formed an individual clade with *S*. *cameli* in the one-humped camel.

Phylogenetic analysis based on the *18S* rDNA, *28S* rDNA, and mitochondrial *cox*1 sequences of *S*. *masoni* confirmed their association with *Sarcocystis* species (Figure 4). In the tree inferred from *18S* rDNA sequences (Figure 4a), *S*. *masoni* in SACs and *S*. *cameli* in the one-humped camel formed an individual clade within a group comprising *Sarcocystis* spp. in ruminants with felids or canids as known or suspected definitive hosts. Based on *28S* rDNA sequences, the phylogenetic tree (Figure 4b) showed that the newly obtained *S*. *masoni* formed an individual clade within a group comprising *Sarcocystis* spp. in ruminants with felids or canids as known or suspected definitive hosts. The phylogenetic tree inferred from mitochondrial *cox*1 (Figure 4c) showed that the newly obtained *S*. *masoni* formed an individual clade with *S*. *cameli* basal to a group comprising *Sarcocystis* spp. in ruminants with canids as known or suspected definitive hosts.

## 4. Discussion

*Sarcocystis* infection is common in many species of animal worldwide, including in China. Sarcocystosis of alpacas has mostly been reported in South America and Australia [1], but recently, the disease was also diagnosed in alpacas from China [22]. Sarcocysts are common in asymptomatic alpacas and other similar Camelidae, usually causing subclinical infections in SACs [4]. An extensive survey in Southern Bolivia indicated that carcass downgrades caused by *Sarcocystis* infection in llamas resulted in 13–20% economic loss to the local farmers [23].

The ultrastructure of the sarcocyst wall is a useful indicator used to distinguish *Sarcocystis* spp. within a given host. A recent review indicated that there are more than 200 *Sarcocystis* species with at least 42 types and several subtypes of sarcocyst wall [1]. Ultrastructurally, the sarcocysts in SACs can be divided into two types. The macroscopic sarcocysts have a cyst wall with cauliflower-like protrusions, similar to TEM wall type 21, known as *S*. *aucheniae* [6,12,24]. The microscopic sarcocysts have a cyst wall with conical to cylindrical vp, similar to TEM wall type 9j, known as *S*. *masoni* [6]. In our materials, only microscopic sarcocysts were observed in the alpaca, and they were identified as *S*. *masoni* due to their high similarity to the original morphological descriptions of the parasite [6]. Recently, morphologically similar microcysts were also diagnosed in alpacas from Peru but were named *Sarcocsytis* sp. based on the slight difference in the sizes of sarcocysts and vp compared with those of *S*. *masoni* [25].

The definitive host of *S*. *aucheniae* in guanaco and llama has been experimentally demonstrated to be dogs [11,12]. In the present study, the life cycle of *S*. *masoni* was completed for the first time, and the dog was proven to be the definitive host of the parasite based on experimental infection and PCR-RFLP. The prepatent period of 8–9 days for *S*. *masoni* is shorter than that of 9–16 days for *S*. *aucheniae* [11,12]. It is worth noting that the molecular data on *S*. *aucheniae* oocysts/sporocysts need to be supplemented in the future to address the possibility of mixed *Sarcocystis* infection in SACs.

Nucleotide sequence analysis has proven to be a useful tool for delineating or identifying species of *Sarcocystis* from the same or different hosts, and different genetic markers have shown different levels of intra- or inter-specific sequence diversity; mitochondrial *cox*1 seems to perform better than nuclear genes for distinguishing *Sarcocystis* spp. in ruminants [13,14,15,20]. To date, there are only *18S* rDNA sequences of *S*. *aucheniae* and *S*. *masoni* deposited in GenBank as references. Here, four genetic markers (*18S* rDNA, *28S* rDNA, *ITS*, and mitochondrial *cox*1) of *S*. *masoni* were sequenced and analyzed. Comparing the newly obtained sequences with those deposited in GenBank, the *18S* rDNA sequences showed 99.4–100% identity with those of *S*. *masoni* obtained from SACs. However, at this locus, they only showed 87.1–91.8% identity with those of *S*. *aucheniae*. Therefore, the *18S* rDNA was suitable for distinguishing *S*. *masoni* from *S*. *aucheniae*. However, the newly obtained sequences of *18S* rDNA and mitochondrial *cox*1 exhibited high similarity with those of *S*. *cameli* from one-humped camels, i.e., 99.6–100% and 98.2–98.5% identity, respectively. Interestingly, *S. cameli* (synonym *S*. *camelicanis*) sarcocysts and *S*. *masoni* sarcocysts present similar morphological characteristics [1,26,27], and the definitive hosts of *S. cameli* are also dogs with a prepatent period of 11 days [26]. Phylogenetic analysis based on the *18S* rDNA or mitochondrial *cox*1 sequences demonstrated that the close relationship between *S*. *masoni* and *S*. *cameli* for both species formed an individual clade in the phylogenetic trees. Therefore, the plausible explanation is that they probably represent the same species of *Sarcocystis* in different hosts. However, this must be proven using more molecular markers of the two parasites and cross-transmission of *Sarcocystis* between SACs and the one-humped camel in the future.

## 5. Conclusions

In summary, based on LM and TEM morphological analysis, only *S*. *masoni* was identified in the alpaca. Four genetic markers (*18S* rDNA, *28S* rDNA, *ITS*, and mitochondrial *cox*1) of *S*. *masoni* were sequenced and characterized. Among them, the sequences of *28S* rDNA, *ITS*, and mitochondrial *cox*1 constituted the first records of the parasite in GenBank. For the first time, the life cycle of *S*. *masoni* was completed, and dogs are its definitive hosts as proven by experimental animal infection and molecular data. In our analysis, the *18S* rDNA and mitochondrial *cox*1 sequences could not effectively distinguish *S*. *masoni* from *S*. *cameli*. The two parasites have a similar LM and TEM morphology and life cycle. Therefore, the relationship between the two species should be further clarified in the future.

## Figures and Tables

**Table 1:** Primers used for the amplification of four DNA regions.

DNA Region	Primer Name	Primer Sequence (5′–3′)	References
*18S* rDNA	ERIB1 ^a^	ACCTGGTTGATCCTGCCAG	[16]
	S2 ^b^	CTGATCGTCTTCGAGCCCCTA	[17]
	S3 ^a^	TTGTTAAAGACGAACTACTGCG	[17]
	B ^b^	GATCCTTCTGCAGGTTCACCTAC	[18]
*28S* rDNA	KL1 ^a^	TACCCGCTGAACTTAAGC	[19]
	KL3 ^b^	CCACCAAGATCTGCACTAG	[19]
	KL6a ^a^	GGATTGGCTCTGAGGG	[19]
	KL2 ^b^	ACTTAGAGGCGTTCAGTC	[19]
	KL4 ^a^	AGCAGGACGGTGGTCATG	[19]
	KL5b ^b^	CTCAAGCTCAACAGGGTC	[19]
*ITS*	ITSF ^a^	GGAATGGAAAGTTTTGTGA	This study
	ITSR ^b^	TTTCTTCTCCTCCGCTTA	This study
*cox*1	SF1 ^a^	ATGGCGTACAACAATCATAAAGAA	[13]
	SR9 ^b^	ATATCCATACCRCCATTGCCCAT	[14]

^a^ Forward primer; ^b^ Reverse primer. The forward and reverse primers for *ITS* used in this study were separately designed using OLIGO 5.0 (National BioScience, Plymouth, MN, USA) based on the newly obtained sequences of *18S* rDNA and *28S* rDNA.

## Data Availability

Not applicable.
